# Smart Wheelchair and Sensor System for Tracking Performance and Accessibility in Urban Environments

**DOI:** 10.3390/s26092657

**Published:** 2026-04-24

**Authors:** Franz Konstantin Fuss, Adin Ming Tan, Oren Tirosh, Yehuda Weizman

**Affiliations:** 1 Chair of Biomechanics, Faculty of Engineering Science, University of Bayreuth, D-95447 Bayreuth, Germany; adintan@gmail.com (A.M.T.); yehuda.weizman@uni-bayreuth.de (Y.W.); 2 Division of Biomechatronics, Fraunhofer Institute for Manufacturing Engineering and Automation IPA, D-95447 Bayreuth, Germany; 3 School of Health and Biomedical Sciences, RMIT University, Bundoora, VIC 3083, Australia; oren.tirosh@rmit.edu.au; 4 Department of Paediatrics, The University of Melbourne, Melbourne, VIC 3052, Australia; 5 Murdoch Children’s Research Institute (MCRI), Melbourne, VIC 3052, Australia

**Keywords:** wheelchair biomechanics, real-world kinematic analysis, inertial measurement unit (IMU), pressure sensing seat mat, assistive technology, propulsion, velocity, GPS, centre of pressure

## Abstract

Wheelchair users face significant mobility limitations related to both medical issues (e.g., musculoskeletal strain, pressure ulcers) and urban accessibility challenges. This pilot study introduces a sensor system integrating an inertial measurement unit (IMU), GPS (Global Positioning System), and a pressure-measuring seat to monitor distance travelled, speed, and posture in relation to real-world conditions. Seven participants navigated an approximately 800-metre outdoor course, divided into 13 sections, while real-time data were recorded. The results showed an average speed of 1.24 ± 0.41 m/s with peak speeds of up to 2.67 m/s. The centre of pressure on the seat fluctuated by an average of 25 mm in the x and y directions (left-right: COPx, back-forward: COPy). The data for average speed, COPx, and COPy showed significant differences between most of the 13 sections, with large, very large, and huge effect sizes. Comparing the speed, COPx, and COPy data with respect to distance travelled, and correlating them between the seven participants by applying the rank-sum method to the mean R^2^ and calculating Kendall’s W, revealed that speed, COPx, and COPy were influenced by course conditions (R^2^ medians between 0.013 and 0.499; W = 0.7857, strong agreement; χ^2^ *p* = 0.0281). Small R^2^ values indicate more individualised participant behaviour, while large R^2^ values highlight the stronger influence of course conditions on the parameters. This non-invasive and cost-effective system provides objective motion data that can be used for future research in wheelchair design and rehabilitation strategies. Despite its advantages, this study was limited to able-bodied participants, so further clinical trials with individuals with mobility impairments are needed.

## 1. Introduction

As reported in 2025, the World Health Organization (WHO) estimated that approximately 80 million people worldwide rely on wheelchairs for mobility [1]. Manual wheelchairs serve as essential assistive devices (AD), enabling individuals with physical disabilities to perform daily activities and maintain functional independence. However, prolonged wheelchair use is associated with several adverse effects, including musculoskeletal disorders, pressure ulcers and biomechanical strain, all of which can significantly diminish users’ quality of life [2,3,4,5,6,7,8]. Moreover, mobility-impaired individuals frequently encounter transportation barriers that hinder their social participation, autonomy, and overall well-being [9,10]. The lack of accessible infrastructure such as steep ramps, damaged sidewalks, obstructed crossings, and poorly designed pathways further exacerbates mobility challenges, limiting users’ ability to navigate their environment safely and efficiently [11].

In recent years, the growing demand for advanced mobility solutions has led to significant innovations in wheelchair technology. This progress is particularly evident in the integration of sensor-based solutions, which facilitate monitoring users’ movement and related parameters in real-world environments. Recent advancements in technology, specifically computer science and artificial intelligence research, have significantly accelerated the development of smart wheelchair studies [12]. Notable progress has been made with Internet of Things (IoT)-enabled sensor systems that evaluate wheelchair functionality and offer real-time biomechanical feedback to enhance users’ mobility.

Many studies have concentrated on assessing kinematics and posture in wheelchair users, highlighting their significance in mobility health, rehabilitation, and injury prevention [13,14,15,16,17,18,19,20,21,22,23,24]. These assessments offer critical insights into biomechanical movement patterns, seating ergonomics, and propulsion efficiency, contributing to improved wheelchair design, user comfort, and long-term musculoskeletal health. Additionally, various studies have examined the practicality and accuracy of sensor-based systems for analysing motion and posture, offering objective and dependable methods for monitoring wheelchair biomechanics [25,26,27].

Sensor-based technologies are vital for monitoring wheelchairs, with inertial measurement units (IMUs) providing a promising method for analysing propulsion dynamics. Research has shown that clinically relevant wheelchair mobility metrics can be reliably collected in real-world settings using one or two IMUs attached to a wheelchair [27]. A recent study [25] investigated the use of hand-mounted IMUs in analysing temporal parameters in manual wheelchair propulsion of users with varying levels of mobility, strength, and experience. IMUs effectively detected hand contact and release events, showing timing errors of ±10 ms for push duration and ±20 ms for recovery duration. They offer a portable, cost-effective, and user-friendly alternative to traditional motion analysis systems, aiding health monitoring in wheelchair users. Lastly, a study by Popp et al. [16] validated the feasibility of IMUs for tracking wheelchair-related activities in both clinical and free-living environments. The researchers evaluated the ReSense device using three computational models: a multi-linear regression algorithm to estimate energy expenditure, a k-nearest neighbours (k-NN) classifier for activity recognition (achieving 97.9% classification accuracy), and an artificial neural network (ANN) model for energy expenditure estimation (yielding a 14.4% error relative to indirect calorimetry). These results underscore the potential of IMU-based systems for monitoring physical activity and metabolic demands in wheelchair users.

Pressure sensors offer important insights for posture assessment, particularly for wheelchair users who are at risk of developing pressure ulcers due to prolonged static positioning. The latter affects blood circulation and induces shear forces at vulnerable areas such as the sacrum and ischial tuberosities [28,29]. Unlike able-bodied individuals, who make frequent postural adjustments implicitly, wheelchair users may struggle to redistribute pressure effectively, increasing their risk of tissue breakdown.

Studies have highlighted the clinical importance of a sensor-based pressure mapping seat for real-time postural assessments and adaptive seating adjustments, with force sensing resistor (FSR) sensors standing out due to their affordability and mechanical flexibility, making them a viable tool for pressure ulcer prevention [13,14,30,31,32]. Yet, despite their practical advantages, FSR sensors exhibit limitations such as signal drift, material fatigue, and lower precision relative to alternative pressure-sensing technologies [33].

Furthermore, although traditional postural assessments are often conducted under static laboratory conditions, they neglect real-world challenges such as uneven terrain, speed variations, and environmental perturbations [24]. Moreover, existing tracking systems often lack integration across multiple biomechanical variables, limiting their ability to assess combined propulsion, posture, and environmental adaptations.

The literature on smart wheelchairs instrumented with multiple sensors is scarce, particularly when specifically searching for publications focusing on wheelchair speed measured with wheel-mounted gyroscopes, seat centre of pressure (COP) movements measured with pressure sensitive mats, and trajectory tracking using GPS signals. The latter implies that experiments must be conducted outdoors. The limited research papers available focus either on navigation and mobility using GPS or gyroscopes, or on postural health using pressure-sensitive mats.

Sundaram et al. [34] investigated seat COP movements to identify pressure-relieving manoeuvres using bending beam force transducers on the wheelchair frame. Pressure-relieving manoeuvres are crucial for mitigating the effects of pressure injuries. Other publications have used three to eight FSR (force sensing resistors) to assess sitting positions and posture [24,35,36,37].

Ohashi et al. [38] investigated the vibrations caused by road surfaces using accelerometers and gyroscopes at constant speed along an outdoor test track recorded by GPS. The data allowed for the differentiation between asphalt interlocking concrete paving stones.

Perez et al. [24] used several sensors on a motorised wheelchair, including proximity sensors as a wheel encoder for distance measurement (and calculation of linear velocity from distance and tests duration); FSRs on the seat and backrest to measure force and position of COP; IMUs, especially gyroscopes for turns and ramp detection; and accelerometer vibration and elevator detection, as well as temperature and humidity sensors. Perez et al. [24] tested the instrumented wheelchair in six different defined tests indoors and outdoors, straight line travel, turns, inclines, roadways and obstacles, free travel and elevator rides.

Cui et al. [39] developed an autonomous wheelchair equipped with GPS, IMU, and 3D LiDAR sensors to enable highly precise positioning route planning and obstacle avoidance. The autonomous wheelchair was tested in an urban environment but without human participants.

The research gap resulting from the accessible literature is that there is no single publication available on smart wheelchairs instrumented with three types of sensors: gyroscopes, pressure-sensitive mats, and a GPS receiver. Such a smart wheelchair should be tested outdoors with human participants in an urban environment and under real-world conditions. This means that the smart wheelchair should be tested on a continuous route with traffic and pedestrians rather than in staged test events, preferably on busy main roads and in quiet residential areas.

The novelty and contribution of this research with an instrumented chair to the specialist literature lie in answering the research questions of what speed and what position of the centre of pressure (COP) are measured on which section of the travel route and to what extent speed and COP position correlate with the environmental conditions. The research gap identified further highlights the need for continuous monitoring approaches to capture dynamic postural adaptations to external factors, such as uneven terrain (e.g., cobblestone pathways), inclines, velocity changes, and peak traffic conditions. A more comprehensive monitoring framework is necessary to assess how sudden accelerations, fluctuations, turns, and CoP movements influence wheelchair posture in outdoor urban environments.

This pilot study aims to develop a cost-effective smart wheelchair with an integrated system using IMUs, GPS, and seat pressure mapping to assess propulsion dynamics and posture. By advancing wheelchair biomechanics, this research seeks to enhance evidence-based mobility strategies, improve user comfort, and support future design innovations.

## 2. Materials and Methods

### 2.1. Participants

Seven able-bodied male participants participated in this study to establish baseline biomechanical trends without confounding variables introduced by pre-existing mobility impairments, comparable to a recent study [24] with five participants. The participants had a mean age of 41.14 ± 8.91 years, a mean mass of 78.57 ± 10.69 kg, and a mean height of 1.76 ± 0.08 m. The participants’ experience with wheelchair propulsion ranged from novice to extensive, with the latter gained from prior research activities. The term “wheelchair user” is used in this publication to refer to physically healthy participants who use a wheelchair as part of research activities.

**Figure 1:**
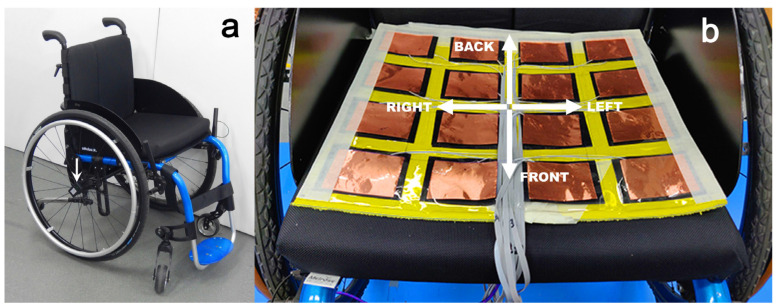
Wheelchair (**a**), and smart seat mat (**b**) used in this study; the white arrow in subfigure (**a**) indicates the position of the wheel sensors.

The data collection involved IMUs for kinematic evaluation, a GPS tracker for trajectory analysis, and an in-house developed pressure mapping seat mat to monitor posture changes and movements during wheelchair propulsion. This study was conducted during peak hours in a suburban area of Melbourne, Australia, on a manual Otto Bock Ventus wheelchair (Figure 1a). Participants travelled an average distance of 787 metres on a defined urban outdoor trail. Ethics approval was granted by the Human Ethics Committee of Swinburne University (approval no. 2019/106) in adherence with the Declaration of Helsinki.

The reasons for recruiting able-bodied male participants for this study can be explained as follows. This project served as a pilot and feasibility study to test the sensor system and the data analysis method. The focus was on determining basic trends such as speed and movement of the centre of pressure, as well as testing the sensor system under controlled and safe conditions before testing it with wheelchair users. For example, users of manual wheelchairs drive individually customised wheelchairs with different seat dimensions, but also different track widths, wheel diameters, and camber angles. To create comparable conditions, we used only one specific wheelchair (Figure 1). This wheelchair may not be suitable for wheelchair users who prefer their own custom wheelchair. The aim of the study was not to compare the driving characteristics of women and men in wheelchairs but rather to analyse the sensor data from different sections of a route with varying conditions.

### 2.2. Instrumentation: IMUs, GPS, and Smart Seat Mat

The wheelchair [40] had 24-inch wheels (diameter 0.61 m, radius 0.305 m) with a camber angle *θ* of 3.2° and a track width of 0.56 m. The chair was equipped with three IMUs (3-Space™ Wireless 2.4 GHz DSSS, Yost Labs, Portsmouth, OH, USA; Figure 1a,b) mounted on the hub of both wheels and beneath the footrest to capture angular velocity data. The angular velocity was recorded at a frequency of 100 Hz, and the gyroscope range was set to 2000 °/s at 16-bit resolution [40]. The data were wirelessly transmitted to a receiver (3-Space^TM^ Wireless Dongle, Yost Labs, Portsmouth, OH, USA), connected to a laptop with a USB cable and placed on the participants’ laps.

For GPS data tracking, we used an android smartphone to collect real-time latitude and longitude position data (1 Hz), which was recorded using the MATLAB mobile app (v 4.9.1, MathWorks, Natick, MA, USA). The data were used to monitor movement paths and distances, as well as to track travel routes. The mobile phone was stored in the rear pocket of the wheelchair backrest.

The in-house developed pressure mapping seat mat (Figure 1b) had a size of 300 × 350 mm and comprised a total of 16 piezoresistive sensor cells. The sensor cells were arranged in a 4 × 4 array, with a cell size of 50 × 65 mm, separated by a 20 mm gap. The sensors were made from off-the-shelf 0.2 mm thick piezoresistive material (Velostat, 3M, St. Paul, MN, USA) [41,42]. The sensor material was placed between two sheets of copper foil (Figure 1b). The piezoresistive sensors were individually connected to 16 reference resistors (70 Ω) in series, and the voltage drop was measured across the reference resistors with a programmable Teensy 3.6 microcontroller (PJRC, Sherwood, OR, USA). The data were collected at 100 Hz and stored on an external SD card break-out board.

### 2.3. Experimental Protocol

Participants moved the wheelchair along a predefined route of approximately 800 m, while data from all systems was recorded simultaneously. To synchronise the IMU and the smart seat mat data, participants simultaneously struck the right and left wheels vertically five times while standing at the beginning and end of the experiment.

The course was selected to evaluate performance under various propulsion conditions, including street turns, uphill and downhill inclinations, and manoeuvring movements over uneven surfaces. These experiments took place on the sidewalks of suburban streets and, where sidewalks were unavailable, directly on the streets (with occasional traffic) during rush hour. To ensure repeatability and consistency, the experiments were conducted under standard conditions.

The course began on the carpark behind Building L6 of Swinburne University (at 6 Luton Lane, Hawthorn, Melbourne, Australia), where the Medical and Sports Engineering Laboratory of Swinburne University was located. Participants then proceeded clockwise along Manningtree Road (slightly uphill), Guest Street (downhill), Burwood Road, Cook Street, Luton Lane (last section steeply uphill), Glenferrie Road (downhill), and back to Manningtree Road. The course ended on the carpark of Building L6.

Before the experiment began, participants were informed about the route (on Google Maps) and its special features such as curves and uphill/downhill sections as well as road and sidewalk traffic.

For safety, at least three researchers (authors of this publication) supervised and accompanied each trial, walking ahead and behind the participant to provide assistance if needed, and to manage traffic and pedestrian interactions, particularly during high-risk sections (larger numbers of pedestrians, oncoming cars, narrow sidewalks, steep inclines, etc.). The participants were instructed to drive at their comfortable speed and to increase their speed when terrain and traffic conditions permitted. The participants and the support staff communicated and alerted each other regarding location and terrain-related features, as well as any intentional speed increases. All participants completed the course unaided.

### 2.4. Data Processing

The three datasets, GPS, IMU, and seat mat, were synchronised as follows. The three IMUs were already synchronised in the first place via the dongle of the sensor system, connected to the laptop. Since the participants simultaneously struck the right and left wheels vertically five times while standing at the beginning and end of the experiment, we identified the ten trigger spikes in the accelerometer signal of the wheel IMUs as well as in the seat mat signal. Synchronisation was achieved by superimposing the ten spikes of both signals, one for the first and one for the last pentads at the beginning and end. The clearest and highest spikes of each pentad were selected, and their timestamps (*t*) were recorded. The data from the measuring mat served as the slave dataset, and the IMU data as the master dataset. The four recorded time stamps were symbolised with *t_Bm_*, *t_Bs_*, *t_Em_*, and *t_Es_*, where the subscripts *B* and *E* denote the start (begin) and end times, respectively, and *m* and *s* denote the master and slave timestamps, respectively. Assuming a linear clock model, with clock offset and skew (i.e., the real-time clocks of the measuring mat and IMU run at different speeds), the slave timestamp *t_s_* was converted into a corresponding master timestamp *t_sm_* as follows:

(1) $$t_{sm}=m\;\left(t_{s}+∆t_{B}\right)+b$$

where *m* and b are the clock skew (slope) and offset (intercept), respectively, and Δt_B_ is the time differential between master and slave timestamps at the first trigger spike:

(2) $$∆t_{B}=t_{Bm}-\;t_{Bs}$$

(3) $$m=\frac{t_{Bm}-t_{Em}}{t_{Bs}-t_{Es}}$$

(4) $$b=\frac{t_{Em}\left(t_{Bs}+∆t_{B}\right)-t_{Bm}\left(t_{Es}+∆t_{B}\right)}{t_{Bs}-t_{Es}}=\frac{t_{Em}t_{Bm}-t_{Bm\;}\left(t_{Es}+t_{Bm\;}-t_{Bs}\right)\;}{t_{Bs}-t_{Es}}\;$$

To complete the synchronisation, the master gyroscope data were assigned to the corresponding *t_sm_* time stamp. Synchronisation ensures a perfect superposition of the selected trigger signals, as their amplitudes were used for the syncing Equations (1)–(4). A synchronisation error can only be determined by analysing all five trigger signals recorded at the beginning and end of the experiment and by their deviations. The standard deviation of 70 data was approximately ±4 centi-seconds. The goal of the synchronisation was not, for example, the precise correlation of speed and COP data, but rather the alignment of both signals on the same timescale, when dividing the total distance into different sections.

To synchronise the IMUs and GPS we aligned the translational velocity calculated from the IMU data (reduced to 1 Hz) and GPS data. The GPS data (latitude and longitude), expressed in micro degrees (µ°), were converted to distances in metres (m) as follows: 10 µ° latitude = 1.11 m in the y-direction and 10 µ° longitude = 0.88 m (1.11 m times cosine of latitude in radians) in the x-direction. The GPS data in metres were superimposed on the satellite images available from Google Maps. The total distance travelled by the participants was calculated from the path defined by the x,y-coordinates of the GPS signal. A rough estimate of speed was obtained by differentiating the distance data in the x- and y-directions with respect to time and calculating the resultant of the x- and y-components of speed. The initial speed increase from a standstill and the final decrease to a standstill were used to fit the GPS speed data to the velocity data calculated from the IMU data. For the latter, the gyro data from the left and right wheels were processed using the method of Fuss [22], which calculates the true wheel speed, frame speed, frame acceleration, distance travelled, turning angular velocity, turning radius, power and energy expenditure. The gyroscopes were calibrated according to the following procedure [43]: the wheelchair equipped with the two gyroscopes was repeatedly moved at different speeds over a total distance of 300 m. The calculated translational velocity of the wheelchair frame was integrated over time and a distance travelled was determined. This distance was compared with the actual distance travelled (300 m). The calculated distance was overestimated by an average of 0.4 m (0.133%), meaning that the instantaneous velocity was also overestimated by the same percentage. To account for the small error, a correction factor was applied to the velocity profiles.

The data from the 16 sensors of the smart seat mat, stored in ASCII format, were converted to the voltage drop across the reference resistors. This voltage drop was then converted to the force exerted on the sensor using individual calibration curves determined using the method of Fuss et al. [41,42]. These calibration curves were generated by repeatedly loading and unloading the sensors (approximately 20 times) between 200 Pa and 0.33 MPa. After determining the peak pressure and conductance data for each loading cycle, these were fitted using a 6th order polynomial to create the calibration curve. The position of the instantaneous centre of pressure (COPx and COPy) on the seat mat was calculated from the weighted average of the x- and y-coordinates of the sensor centroids, weighted by the force of each sensor.

**Figure 2:**
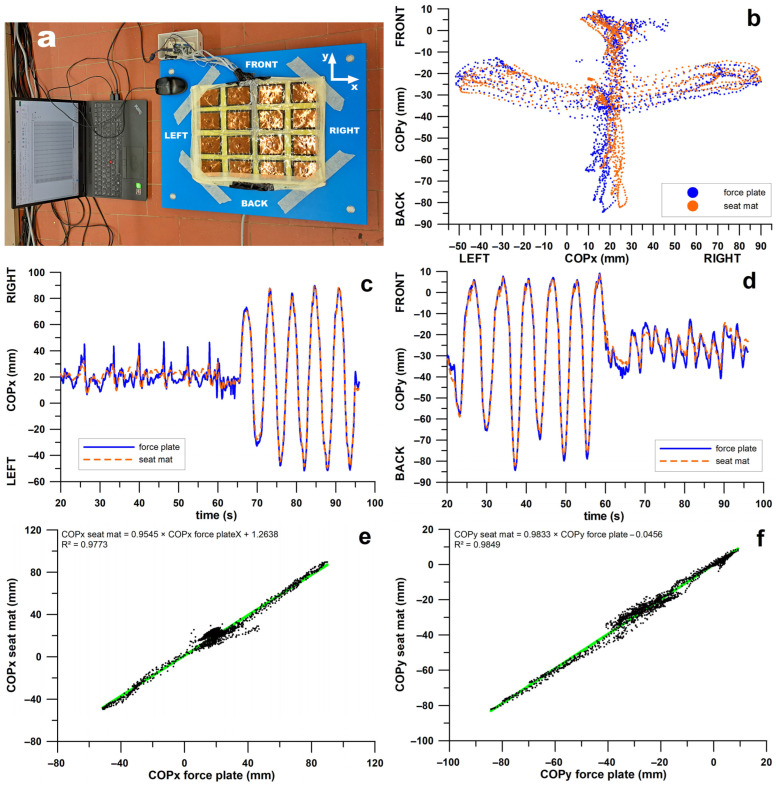
(**a**) Test set-up for validating the centre of pressure (COP) of the seat mat, mounted on a force plate; (**b**) COPy vs. COPx; (**c**,**d**) COPx and COPy vs. time; (**e**,**f**) correlations of COP seat mat vs. COP force plate; green: regression line.

The COP calculated from the seat mat was validated using an independent, non-wearable gold standard: a portable force plate with four triaxial piezoelectric sensors (Kistler 9260BA6; Winterthur, Switzerland). Validation was performed analogously to the validation of a smart insole [44]. The seat mat was attached to the force plate (Figure 2a). One author of the present study sat on the mat and moved his upper body forward/backward and left/right. The force plate and seat mat data were synchronised as described above. After aligning the centre point coordinates of seat mat and force plate, the COPs were superimposed by plotting COPy vs. COPx (Figure 2b), and COPx and COPy vs. time (Figure 2c,d), to assess the similarity of the movement patterns. Subsequently, the COPs of seat mat and force plate were correlated (Figure 2e,f) to assess whether the COP data for the seat mat and force plate showed similar trends.

### 2.5. Statistics

To capture the specific characteristics and properties of the course, it was divided into 13 sections. For each participant, the averages of speed and positions of COPx and COPy in each section were compared individually using a one-way ANOVA test. From 13 groups (i.e., sections) 78 pairs were generated, which were compared using the Tukey-HSD post hoc test. Additionally, the effect size (Cohen’s d) was calculated for each pair. To visualise which pairs showed a significant difference in their means and the magnitude of the effect size, a radial network diagram with 13 nodes was used. For clarity, two nodes were connected by a line only if their effect size, averaged across all seven participants, was at least d = 0.8 (large effect size). Line thickness was coded such that the thinnest line corresponds to an average effect size of d = 0.8 and the thickest line to an average effect size of d ≥ 2 (huge effect size). This method only considered large, very large, and huge average effect sizes, while medium, small, and very small effect sizes were excluded for the sake of clarity. We used an N-of-1 approach to capture the high interindividual variability inherent in wheelchair mobility in urban environments. While we acknowledge that pairwise comparisons within a single trial can generate serial correlations (i.e., pseudoreplication), these metrics are used here descriptively to identify individual-specific responses to route conditions (e.g., terrain, traffic, etc.) rather than draw population-level inferences. To minimise the risk of overinterpreting individual-level noise, the radial network graphs display only relationships that, on average across the entire cohort, exceed a threshold of d = 0.8 (large effect size). By showing only connections with an average d ≥ 0.8 across all seven participants, the noise of a single trip is filtered out, and only reproducible biomechanical phenomena are highlighted. This ensures that the analytical depth highlighted by the network graphs represents robust, shared trends across the seven participants.

To assess the individual responses of the seven participants to the course, the speed, as well as the positions and COPx and COPy, were compared using linear regression. For this purpose, the three datasets had to be “syn***chor***ised” (from ancient Greek χ*ῶ*ρος, the space, in contrast to χρόνος, the time), i.e., aligned to space (distance covered over the course). The three datasets, speed, COPx and COPy, share the same timestamp but not the same distance “stamp”. However, the data have to be compared based on the measurement location. Syn***chor***isation was achieved by using Equations (1)–(4), where *t* was replaced by a master distance scale.

Intuitively, one might expect that speeds on a stretch of road would be higher if there is less traffic on the road or sidewalk, or if the road is downhill rather than uphill. Nevertheless, this expectation has to be verified. More importantly, are there differences in the position of the COP (in the x and y directions) caused by environmental conditions?

To investigate these questions, the data from the 21 pairs of participants in the seven groups were correlated, and the R^2^ values and the corresponding *p*-values were calculated (α = 0.1 in regressions). Since the route conditions and characteristics are the only shared variable between the participants, the R^2^ value represents a measure of route influence. For example, an R^2^ value of 0.3 means that 30% of the data from participant A are explained by the data from participant B, and vice versa, based on the road conditions. The six R^2^ values for each participant and each parameter (speed, COPx, and COPy position) were averaged (median) and compared. This comparison clarifies both the individual behaviour of the seven participants and the track conditions. The participant with the highest average R^2^ value reacts particularly strongly to the environment, and their speed directly reflects the terrain, which tells us the most about the track conditions. Conversely, the participant with the smallest average R^2^ value reveals the most about their individual behaviour, which is least dependent on the track conditions. To investigate interindividual variability across the entire route, we conducted a cross-correlation analysis of the speed profiles of all participants. Since the resulting R^2^ matrix contains redundant pairs (e.g., R^2^_P1/P7_ = R^2^_P7/P1_, where P represents the respective individual), we used these values descriptively. Participants were ranked according to their mean R^2^ value to distinguish between those whose speed was primarily determined by the route conditions (high R^2^ value) and those with highly individualised behaviour (low R^2^ value). Thus, our pilot study aims to quantify the range of each participant’s behaviour as a descriptive Route Compliance Index. To ensure the robustness of the three parameters studied (speed, COPx, and COPy values), we performed a rank consistency analysis. This allowed us to identify participants who consistently exhibited individualised behaviour across multiple biomechanical domains. We believe that this descriptive approach more accurately reflects the inferential strength of a pilot sample (*n* = 7) while simultaneously highlighting the crucial interindividual variability. To this end, we applied the rank-sum method to the mean R^2^. Furthermore, we calculated Kendall’s W (coefficient of concordance), which measures the agreement between the three parameters (speed, COPx, and COPy values).

## 3. Results

### 3.1. GPS Data

**Figure 3:**
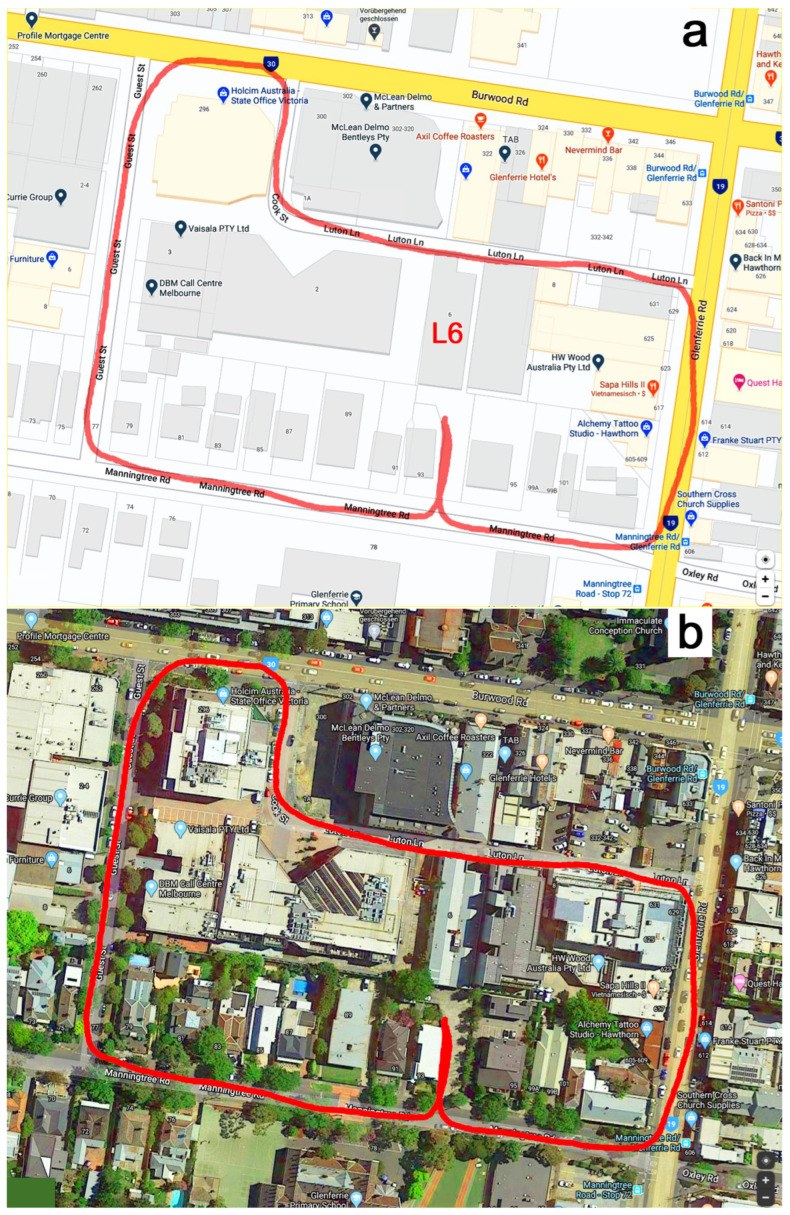
Average GPS path (highlighted in red) superimposed on (**a**) street view and (**b**) satellite view of Google Maps; L6: building at 6 Luton Lane.

The average GPS path covered by the participants (Figure 3) was 786.6 ± 3.4 m (780.7–791.4 m) long. The average travel time was 636 s (10.6 min), corresponding to an average speed of 1.24 m/s (4.45 km/h). The accuracy of the GPS track is not precise, as Figure 3 suggests that participants travelled on the left sidewalk of Guest Street and directly on Burwood and Glenferrie Roads. In fact, participants travelled on the north sidewalk of Manningtree Road, the right sidewalk of Guest Street, the south sidewalk of Burwood Road, and the left sidewalk of Glenferrie Road.

**Figure 4:**
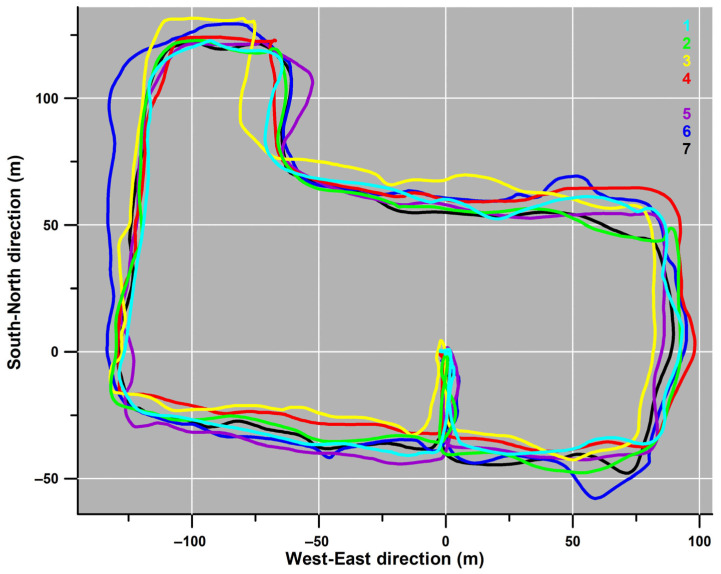
GPS path of all seven (1–7) participants (clockwise direction); start and end of the paths at (0, 0).

Figure 4 shows the individual GPS data of all participants. Although participants travelled the same route, their GPS data were not consistent but similar. For example, participant 6’s data deviated westward on the north side of Guest Street (Figure 4 blue path, x: −125 m, y: 100 m). Compared to the x-direction average (−117.3 ± 1.4 m) for participants 1–5 and 7 at y = 100 m, participant 6’s GPS datum at x = −132.4 m was off by 15.1 m.

### 3.2. Velocity Data from the IMUs

**Figure 5:**
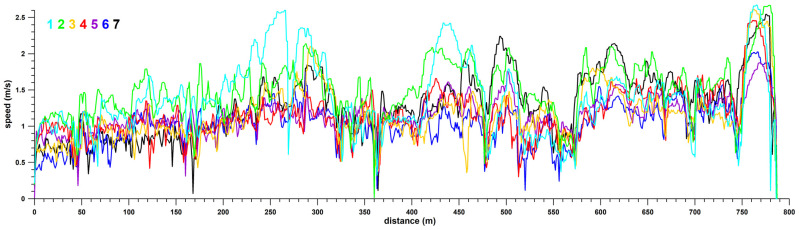
Velocity vs. distance; velocity profiles of all seven (1–7) participants.

**Figure 6:**
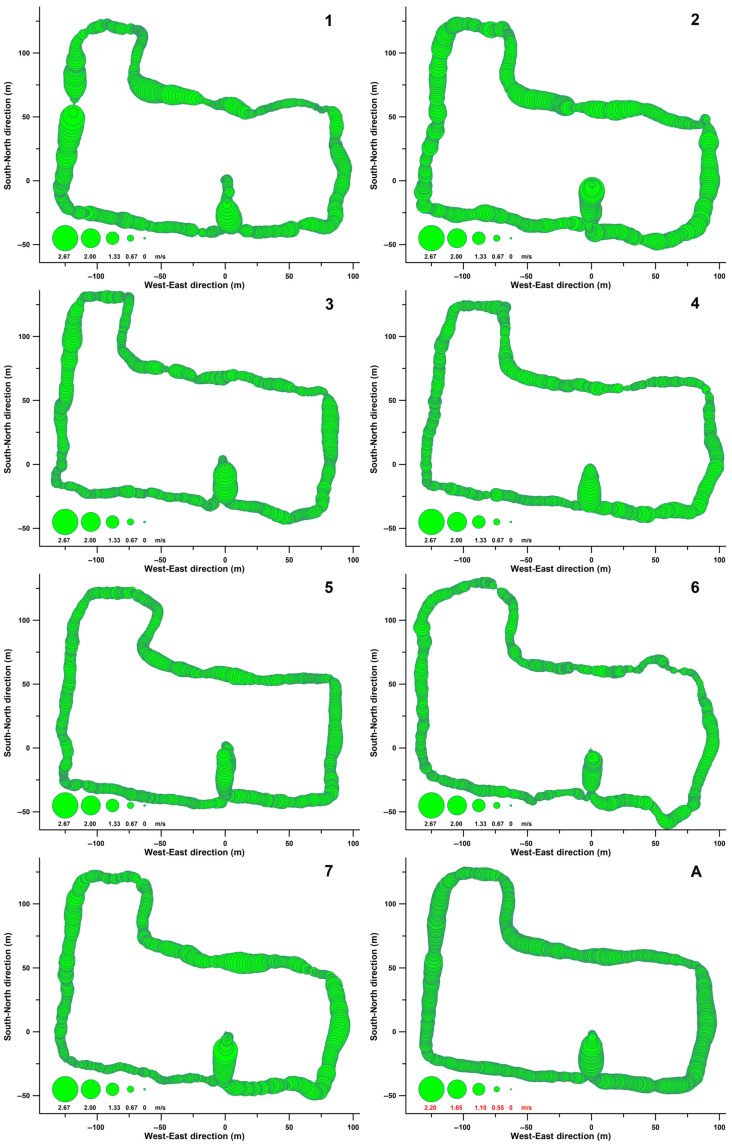
GPS path of all 7 participants (in the subfigures, the numbers in the top right corner refer to the participants’ numbers (**1**–**7**), and the letter **A** in the top right corner of the last subfigure indicates the GPS path averaged across the seven participants); the bubble size corresponds to the wheelchair speed; note that the averaged wheelchair speed data related to the bubble size in subfigure ‘**A**’ (highlighted in red font) are different from those shown in subfigures **1**–**7**.

Figure 5 shows the speed profiles of all seven participants (1–7) versus the distance. Although the trend is quite similar across all participants, individual differences are evident in Figure 6. Figure 6 shows the speed fluctuations relative to the GPS path, suggesting that specific location and terrain characteristics forced participants to slow down. Furthermore, some participants (3, 4, 6, 7) started slowly on Manningtree Road (Figure 3a; x < 0 m, y < −10 m in Figure 4), reflecting their inexperience in wheelchair driving. At the end of the circuit, after turning off Manningtree Road into the car park (at 750 m in Figure 5), the participants attempted one last high-speed run, reaching their maximum speed over the entire distance (participant 1: 2.671 m/s = 9.6 km/h, participant 2: 2.668 m/s, participant 3: 2.608 m/s, participant 7: 2.545 m/s, participant 4: 2.458 m/s, participant 6: 2.024 m/s and participant 5: 1.875 m/s). Except for the final high-speed section, the peak speeds reached (>2.12 m/s) for participant 1 were 2.196, 2.599, and 2.357 m/s on Guest Street (downhill), and 2.425 m/s after turning from Cook Street into Luton Lane (Figure 6(1)); for participant 7, 2.239 m/s in the middle of Luton Lane and 2.139 m/s in the middle of the Glenferrie Road section (downhill; Figure 6(7)); and for participant 2, 2.137 m/s on Guest Street just before turning into Burwood road (Figure 6(2)).

**Figure 7:**
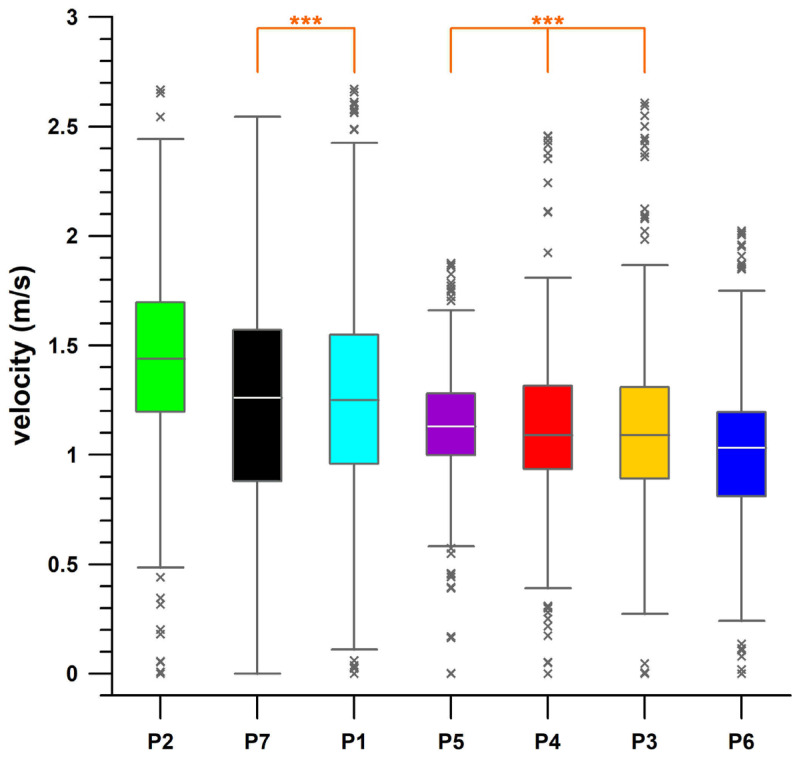
Box plots of the participants’ velocity data; P1–P7: participants 1–7; ×: outliers; ***: ***non***significant differences (*p* > 0.05; Friedman rank sum test, post hoc tests with Conover method).

**Figure 8:**
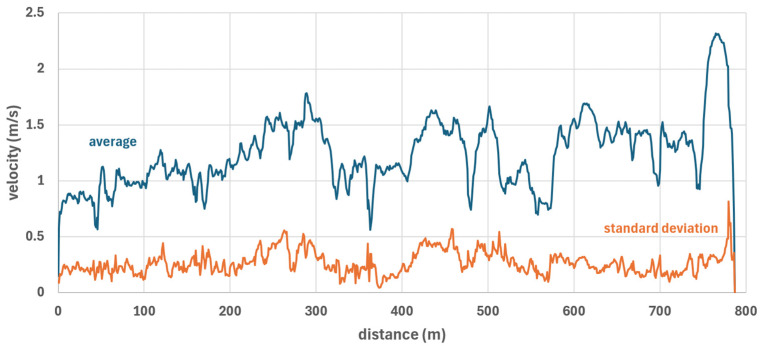
Velocity vs. distance; average speed (blue curve) and standard deviation (orange curve) of all seven participants.

Participant 2 was the fastest with an average speed (Figure 7) of 1.44 m/s (median), followed by participants 7 and 1 (median 1.25) and participants 3, 4, and 5 (median 1.11); participant 6 was the slowest (median 1.03). Figure 8 shows the average speed of all participants and the standard deviation relative to distance. The standard deviation was below 0.5 m/s in almost all instances.

**Figure 9:**
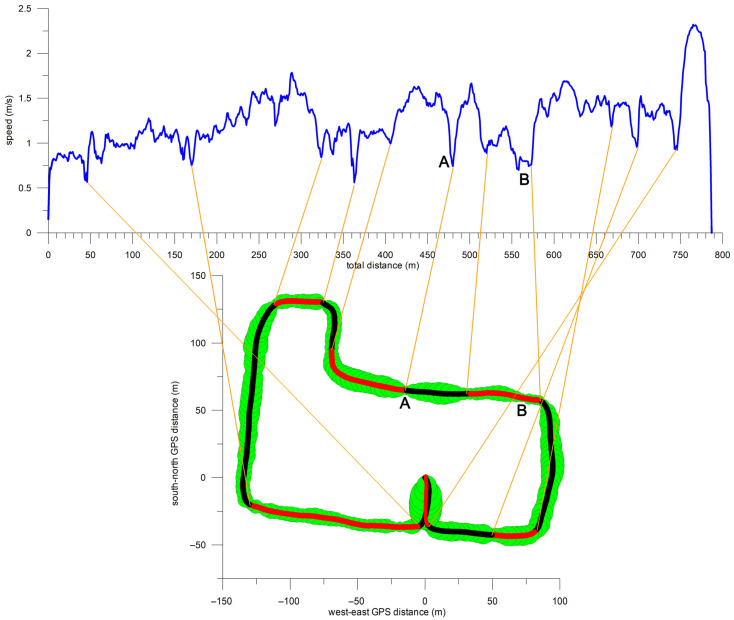
Feature extraction from the correlation of average speed and terrain features; average speed vs. distance (upper plot); different sections of the GPS path (lower plot; bubble size corresponds to speed magnitude); **A** & **B**: reduced speed referring to subfigures **A** and **B** in Figure 10.

**Figure 10:**
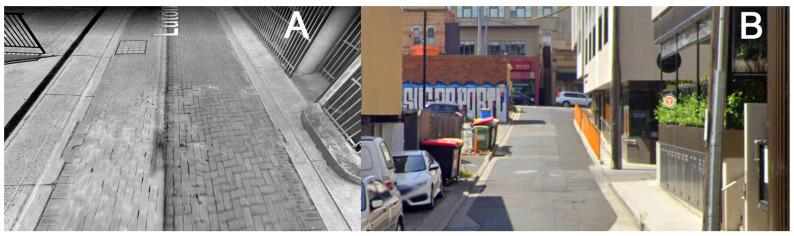
Terrain features identified in Figure 9; (**A**): feature A in Figure 9, defective paving stones in Luton Lane; (**B**): feature B in Figure 9, steep uphill section of Luton Lane before joining Glenferrie Road.

Figure 9 links eleven instances of speed reduction to the location and terrain features of the GPS path. The main reason for the speed reduction was 90° turns (seven instances). Other reasons included the end of the sidewalk on Cook Street just before the turn into Luton Lane; defective paving stones on Luton Lane (‘A’ in Figure 9 and Figure 10A); an increasing gradient in the eastern section of Luton Lane with a steep climb at the end of Luton Lane (‘B’ in Figure 9 and Figure 10B); and uneven sidewalk surface on Manningtree road including a sharp edge between two concrete slabs.

**Figure 11:**
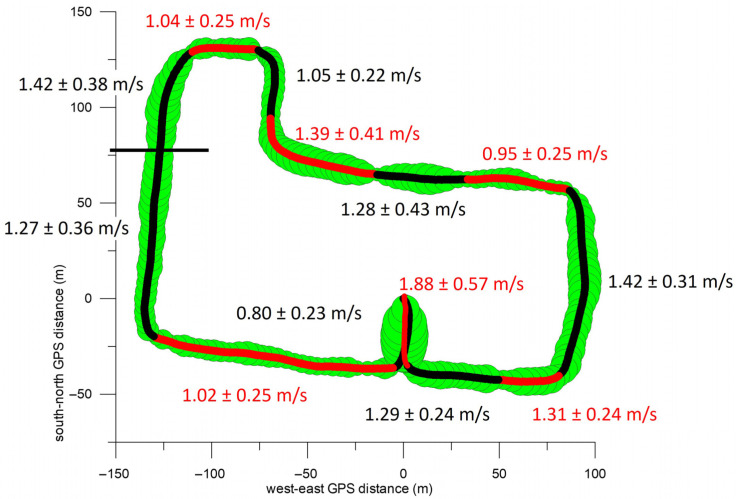
GPS path and sections including the individual velocities (average and standard deviation).

Figure 11 shows the average speeds on the various sections of the route. The highest average speed was reached on the final section of the route, namely in the car park behind the L6 building (1.88 m/s, 6.8 km/h). The second highest speed was recorded on the sidewalks of Guest Street (final third) and Glenferrie Road, each at 1.42 m/s (5.1 km/h). Both roads had a downhill profile. Other sections with high speeds were the first section of Luton Lane (1.39 m/s), the first and second sections of Manningtree Road (1.31 m/s and 1.29 m/s, respectively), the middle section of Luton Lane (1.28 m/s), and the first two-thirds of Guest Street (1.27 m/s, downhill). Low speeds were recorded on the sidewalks of Manningtree Road (eastern section), Burwood Road, and Cook Street (1.02–1.05 m/s). The lowest speeds were recorded on the steep uphill section of Luton Lane (0.95 m/s) and at the beginning of the route (car park, wheelchair familiarisation, 0.8 m/s).

**Figure 12:**
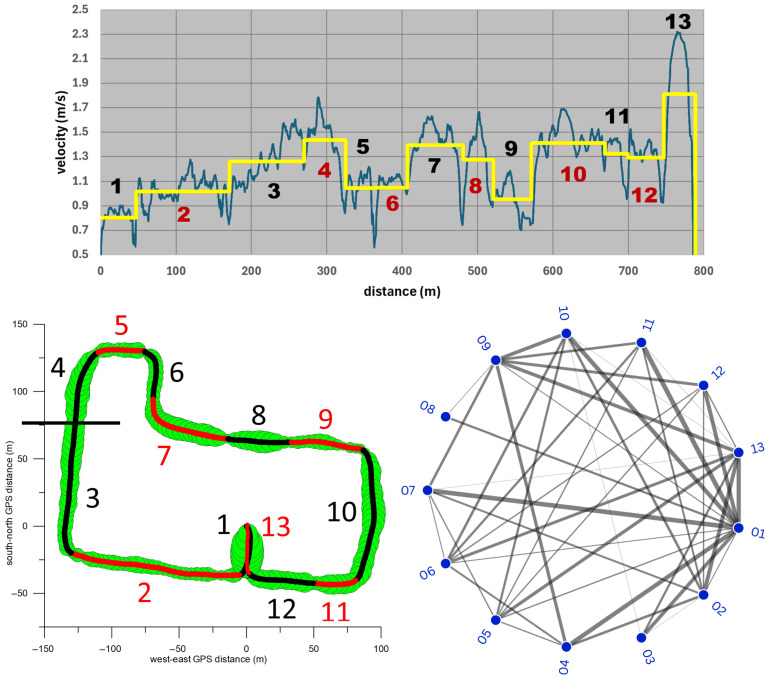
**Top graph:** average speed vs. distance (blue signal) and average speed of the 13 sections (numbered 1–13) of the circuit. **Bottom left:** 13 sections of the GPS path. **Bottom right:** radial network graph. The lines between two nodes (corresponding to a pair of the 13 sections) indicate a significant difference between the respective average speeds; the thickness of the lines indicate the effect size, where the thinnest line corresponds to an effect size of d = 0.8 (averaged across the seven participants), while the thickest line corresponds to an effect size of d ≥ 2.

To assess the extent to which the location and terrain of the route influence the speed in the different sections (Table 1), we compared the speed differences between all sections using the one-way ANOVA test. Figure 12 shows that the average section speed increased significantly from section 1 (car park) through 2 (Manningtree Road) to 3 (first two-thirds of Guest Street, downhill) and 4 (final section of Guest Street, downhill). All three increases had very large, large and medium effect sizes, respectively.

After that, the speed decreased significantly with a very large effect size, and on Burwood Road and Cook Street (sections 5 and 6) there was no significant speed difference. After the turn onto Luton Lane (section 7), the speed increased significantly with a very large effect size, followed by two significant speed reductions with medium and very large effect sizes, respectively (sections 8 and 9). After the steep climb in section 9, the speed increased significantly with a very large effect size. The average speeds on the following sections (10, Glenferrie Road, downhill; 11 and 12, Manningtree Road) did not differ significantly. The speed on the final stage in the parking lot (section 13) differed significantly from that on section 12 with a very large effect size, which was due to the participants’ deliberate increase in speed.

Figure 12 (bottom right) further shows that the speed in section 1 differed significantly from that of all other sections (with at least a large effect size). The same applies to the speed in section 13, with the exception of sections 4 and 8, where the speed difference compared to section 13 had only a medium effect size. Other notable significant speed differences occurred between sections 7 and 9, 6 and 10, 5 and 11, 2 and 10, 4 and 9, 2 and 7, and 4 and 6, all with at least large effect sizes (Figure 12, bottom right). The median of all significant speed differentials was 0.32 m/s (inter quartile range: 0.32 m/s; maximum: 1.32 m/s; minimum 0.05 m/s).

To differentiate between individual behaviour and track conditions as influencing factors on speed, the speed profiles of the seven participants were pairwise correlated, and the six R^2^ values per participant were averaged (median). Participants 2–7 exhibited R^2^_med_ values between 0.44 and 0.50 (Table 2). If a participant’s speed explains 50% of the variance in the speed of another participant, half of this difference is attributable to track conditions and characteristics, as these represent the only common variable among the participants, while the other half is based on individual behaviour. A stronger influence of individual behaviour is evident in participant 1, the most experienced wheelchair user, who therefore likely represents a true outlier (R^2^_med_ < Q1—1.5 IQR, where Q is any quartile, and IQR is the interquartile range, i.e., Q3–Q1) with an R^2^_med_ value of 0.31. The data suggest that approximately ⅓ of participant 1’s speed is influenced by the course, while ⅔ is by individual behaviour. In fact, participant 1 maintained the highest speed of all participants for 27.4% of the course length at an average speed of 1.85 m/s (Figure 5). Participant 2, with the highest average speed (Figure 7) maintained maximum speed over 46.95% of the course length at an average speed of only 1.58 m/s (Figure 5).

### 3.3. Validation Results of the Smart Seat Mat

Figure 2b–d shows that the COPs of the seat mat and force plate follow the same pattern. The validation experiment began with forward and backward movements (y-direction, Figure 2d), followed by lateral movements (x-direction, Figure 2c). In addition to the larger movements in one direction, very small movements also occurred in the other. In the x-direction, these small movements (Figure 2c) showed the same trend, but the COPx of the force plate had a larger amplitude. In the y-direction, the small movements (Figure 2d) also showed the same trend, but the COPy of the force plate had only a slightly higher amplitude. The COPs of the larger movements were almost perfectly matched.

Figure 2e,f shows the correlations of seat mat vs. force plate data. The regression equation (Figure 2e,f) ideally reads Y = 1 × X + 0. The shallower slope of the equations in Figure 2e,f indicates that the COPs of the seat mat have a slightly smaller amplitude than those of the force plate. The intercept shows that the COPx of the seat mat is shifted an average of 1.26 mm further to the right compared to the force plate. In contrast, the COPy of the seat mat is shifted only slightly, by 0.05 mm, to the rear.

The Pearson R^2^ shows that the COPx of the force plate explains 97.7% of the variance of the COPx of the seat mat; the COPy of the force plate explains 98.5% of the variance of the COPy of the seat mat. Since the Spearman R^2^ value was smaller than the Pearson R^2^ value in both the COPx model (0.7908) and the COPy model (0.9711), both correlations (Figure 2e,f) are nearly linear. The relatively low Spearman R^2^ value for COPx (0.7908) is due to a high-density cluster, which is clearly visible in the centre of the point cloud in Figure 2e.

### 3.4. X-Direction of Centre of Pressure Data from the Smart Seat Mat

**Figure 13:**
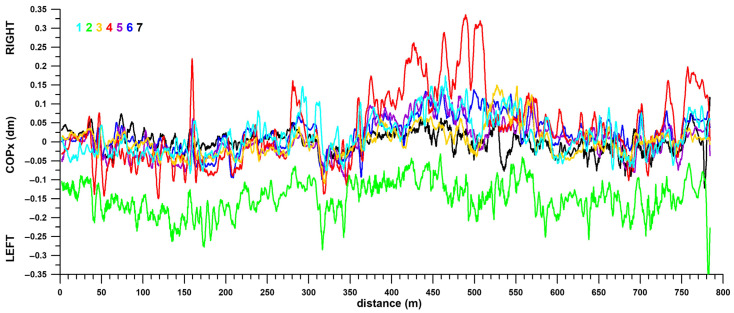
COPx vs. distance; COPx data of all seven (1–7) participants averaged over 4 m.

Figure 13 shows the fluctuations of the centre of pressure in the x-direction (COPx, right-left) over distance. The COPx fluctuated by approximately 10–15 mm around the centreline. These deviations appear small, but the trend and pattern across all participants are quite comparable. Even individual peaks are repeated across participants. These observations also apply to participant 2, who was consistently seated off-centre and whose COPx shifted approximately 150 mm to the left.

Participant 4’s COPx occasionally shifted to the right. The first sharp peak at 160 m coincided with the right-hand turn from Manningtree Road onto Guest Street. Four more prominent and broad peaks occurred between 410 and 520 m, i.e., along the entire length of Luton Lane up to the steep climb. Another broad peak coincided with the final high-speed section in the car park.

**Figure 14:**
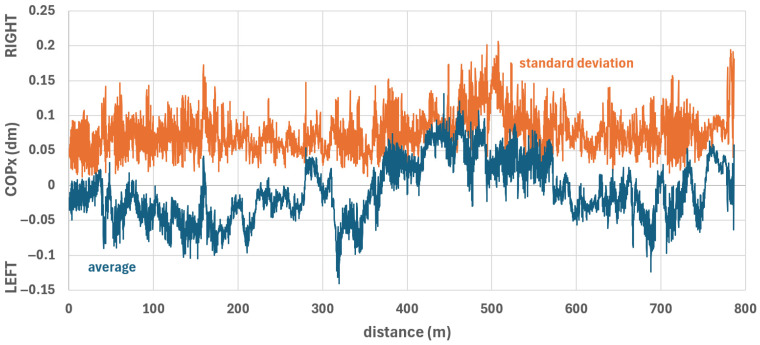
COPx vs. distance; average COPx (blue curve) and standard deviation (orange curve) across all seven participants.

**Figure 15:**
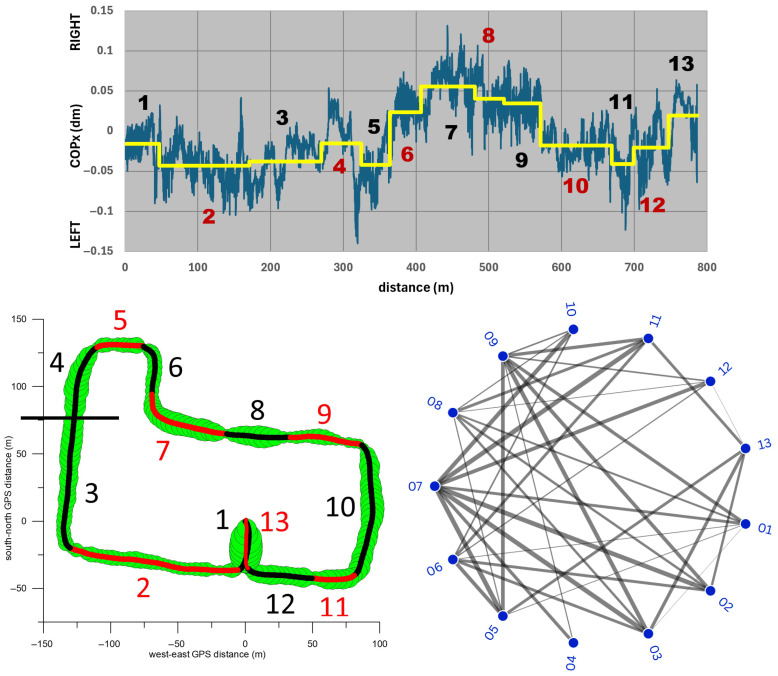
**Top graph:** average COPx vs. distance (blue signal) and average COPx position of the 13 sections (numbered 1–13) of the circuit. **Bottom left:** 13 sections of the GPS path. **Bottom right:** radial network graph. The lines between two nodes (corresponding to a pair of the 13 sections) indicate a significant difference between the respective average COPx positions; the thickness of the lines indicate the effect size, where the thinnest line corresponds to an effect size of d = 0.8 (averaged across the seven participants), while the thickest line corresponds to an effect size of d ≥ 2.

Figure 14 shows the average COPx position of all participants and the standard deviation relative to distance. The standard deviation was smaller than the total COPx fluctuation range. A comparison of the COPx differences across all sections (Figure 15) shows that the COPx variations between the extreme COPx position on the right (section 7) and eight (out of twelve) other sections are significantly different (with at least large effect sizes). The same applies to sections 8 and 9 (second and third extreme COPx positions on the right) and seven and eight additional sections, respectively. On the left, the extreme COPx positions are in sections 2, 3, 5, and 11, with significant differences (with at least large effect sizes) in four, four, six, and four additional sections, respectively. The median of all significant COPx differences was 3.75 mm (interquartile range: 4.50 mm; maximum: 26.93 mm; minimum: 0.39 mm).

Regarding individual behaviour and track conditions influencing COPx positions, participants 1–7 showed R^2^_med_ values between 0.11 and 0.43 (Table 2). In contrast to the R^2^_med_ values for speed, the lowest COPx R^2^_med_ value of 0.11 for participant 7 does not represent an outlier.

### 3.5. Y-Direction of Centre of Pressure Data from the Smart Seat Mat

**Figure 16:**
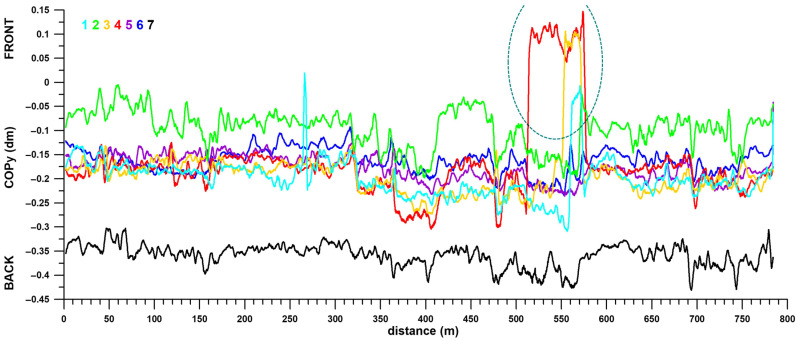
COPx vs. distance; COPx positions of all seven (1–7) participants averaged over 4 m; the dashed ellipse highlights a sudden COPy movement forward.

Figure 16 shows the fluctuations of the centre of pressure in the y-direction (COPy, forward-backward) over the distance. The COPy fluctuated by approximately 1–2 cm (excluding special events). Like the COPx, these fluctuations show the same trend and pattern for all participants. Likewise, individual peaks are repeated for all participants.

**Figure 17:**
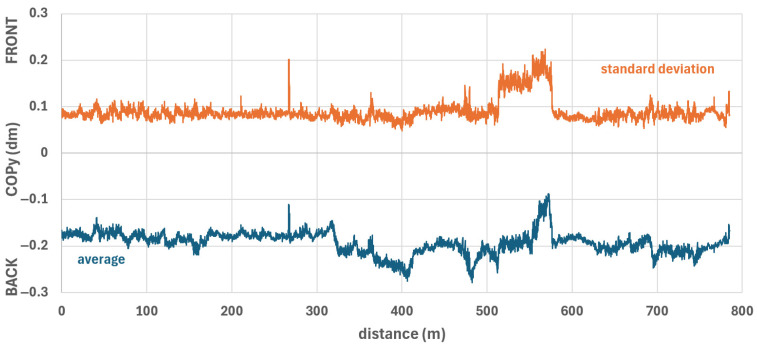
COPy vs. distance; average COPy (blue curve) and standard deviation (orange curve) of all seven participants.

**Figure 18:**
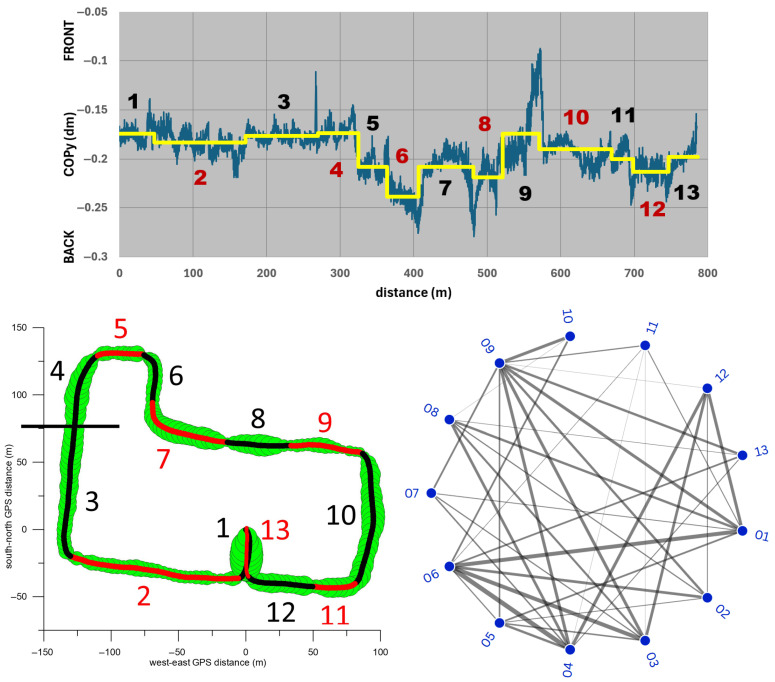
**Top graph:** average COPy vs. distance (blue signal) and average COPy position of the 13 sections (numbered 1–13) of the circuit. **Bottom left:** 13 sections of the GPS path. **Bottom right:** radial network graph. The lines between two nodes (corresponding to a pair of the 13 sections) indicate a significant difference between the respective average COPy positions; the thickness of the lines indicate the effect size, where the thinnest line corresponds to an effect size of d = 0.8 (averaged across the seven participants), while the thickest line corresponds to an effect size of d ≥ 2.

All participants showed a more forward-directed COPy between sudden forward (405 m) and backward movements (480 m) on Luton Lane until the first slight incline. Participant 1’s sharp COPy peak after 260–270 m (Figure 16) coincided with the sudden deceleration of his wheelchair (Figure 5 and Figure 6(1)), which moved his COPy forward. On the steep incline at Luton Lane between 510 and 580 m (dashed ellipse in Figure 16), four participants extended their legs forward to move the COPy in the same direction and thus achieved a better angle of attack for uphill propulsion. Participant 4 started early at 510 m (4 cm COPy movement), followed by participant 3 at 540 m (3.5 cm movement), participant 1 at 560 m (3 cm movement), and participant 2 at 570 m (2 cm movement). The other three participants had no problems with the reduced angle of attack due to the reclined wheelchair when climbing. These two events at 260–270 m and 510 and 580 m are also reflected in the COPy average and standard deviation across all participants (Figure 17). While the COPy standard deviation is less than 1 cm in most instances, it increased to 1.5–2 cm for these two events. An interesting forward movement of COPy can be observed between 400 and 480 m (Figure 16 and Figure 17). This corresponds to the entirety of section 7 (Figure 9 and Figure 18) and the simultaneous increase in speed during this section (Figure 5 and Figure 9). The forward movement of the COPy while seated is due to the forward bending of the upper body, which facilitates better power transfer to the wheels. The COPy movement varied between 2.1 mm (participants 1 and 5) and 12.7 mm (participant 3).

A comparison of the COPy differences across all sections (Figure 18) reveals significant differences in the backward movement of the COPy from section 4 through 5 to 6 (at least very large effect sizes), the forward movement from section 6 to 7 and 8 to 9 (large effect sizes), and the backward movement from section 9 to 10 (huge effect size). Of the sections with the foremost COPy, sections 1, 3, 4, and 9 show significant differences with at least a large effect size compared to 7–10 other sections. Of the sections with the rearmost COPy, sections 6 and 8 show significant differences with at least a large effect size when compared to 6–8 other sections. section 5 shows significant differences with at least a large effect size when compared to 6 other sections, 5 sections with a more forward COPy, and 1 section with a more backward COPy (Figure 18).

Regarding individual behaviour or track conditions influencing COPx positions, participants 1–7 showed R^2^_med_ values between 0.01 and 0.24 (Table 2). These values suggest that COPy position is determined more by individual behaviour than by track conditions.

### 3.6. Influence of Track Conditions on the Participant Speed and COPx and COPy Position

In this pilot cohort, we observed a wide spectrum of route compliance, with median coefficients of determination (R^2^) ranging between 0.013 and 0.499 (Table 2). Although the small sample size does not allow for definitive inferential partitioning, this range indicates that route conditions do not have a uniform effect on all users. Kendall’s W = 0.7857 (χ^2^ *p* = 0.0281), calculated from Table 3, indicates strong agreement, meaning the participants were highly consistent regarding how much they were influenced by the route across all three parameters. Participant 3 is consistently the most individualistic (rank sum 7), while participant 5 is consistently the most route-driven (rank sum 21; Table 3). These two individuals represent the phenomenological boundaries of our study. Even in a small sample, the identification of such stable, multiparametric behavioural extremes is a robust finding. Regarding the IQR values, P6 (low speed, IQR: 0.021) is not only route-driven but also highly predictable. His low IQR value suggests that his behaviour is extremely stable across the 13 sections. P7 (high speed, IQR: 0.098) was in the middle range of individual behavioural consistency for rank but had a very high IQR value. This means he is an adaptive driver: in some sections he is highly route-driven, while in others he drives very independently. The interquartile range (IQR) demonstrates that our small sample does indeed reveal complex and distinct behavioural patterns.

**Figure 19:**
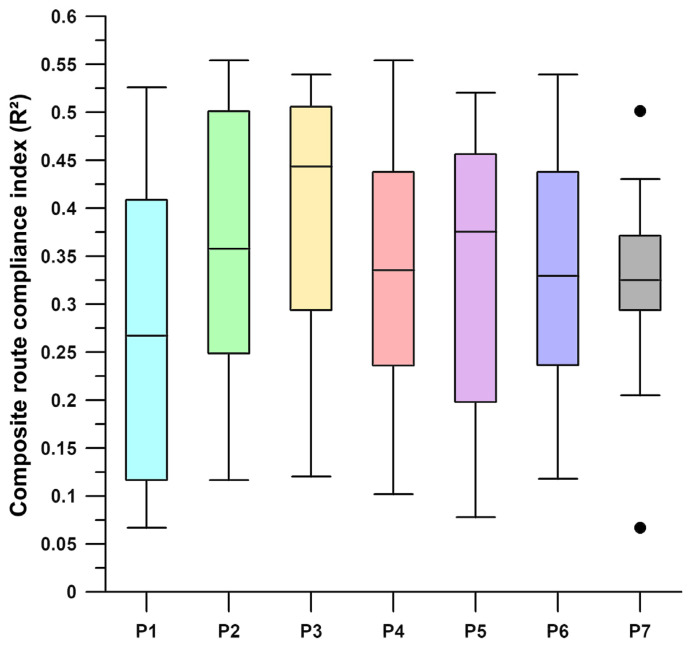
Composite distribution of route-compliance coefficients (R^2^) across participants, box plots of the participants’ R^2^ data; P1–P7: participants 1–7; ●: outliers.

The boxplot in Figure 19 shows behavioural patterns. P3 and P1 have the highest and lowest medians, respectively. The median of P3 is above the 75th percentile of P1. This suggests that P3’s lowest compliance to the route is often higher than P1’s average compliance. P7 has the tightest whiskers and two outliers. This suggests that P7 has a very specific, tight driving style and only deviates from the route’s influence in very specific segments (i.e., the outliers). P1, P2, and P5 have large interquartile ranges (IQRs). These participants are unpredictable: sometimes very route-oriented, sometimes completely individualistic.

To contextualise the seemingly different and contradictory results of the rank-sum method (Table 3) and the box plot (Figure 19), we classify the box plot to define Absolute Compliance, which represents the global magnitude of R^2^, and the rank-sum method (Kendall’s W) was used to determine Domain Consistency, with the ranking reflecting the trait stability. While P1 exhibited the lowest R^2^ magnitude for certain parameters (Figure 19), participant 5 proved to be the most consistent conformist across all biomechanical domains (highest rank sum, Table 3). These results indicate that the influence of the route is a stable factor for some users across both mechanical and physiological control mechanisms.

## 4. Discussion

This study presents, based on the available and accessible literature and to the best of the authors’ knowledge, the first instrumented wheelchair system with an integrated IMU, GPS, and a pressure-measuring seat mat. The novelty of this research lies in the fact that the combined data from these three different sensor systems enable a comprehensive analysis of user kinematics with regard to the following:

- Wheelchair speed and centre of pressure (COP) movements in response to the environment;
- Correlation of speed and COP movements with features and conditions of the route;
- Influence of these features and conditions on the driving behaviour of individual participants with respect to speed and COP movements.

Manual wheelchairs play a crucial role in the mobility of millions of people worldwide. However, prolonged use can contribute to muscle fatigue and overuse syndromes, cardiovascular strain, pressure sores and ulceration, and a range of secondary health problems that can negatively impact the user’s long-term health and quality of life [2,3,4,5,6,7,8].

The main results show that the integration of these sensors enabled a comprehensive understanding of the kinematics and postural adjustments during locomotion in the open, with a focus on the dynamic responses to terrain variations. The recorded wheelchair average speed showed substantial variability among participants, with an average speed of 1.24 ± 0.41 m/s and maximum average speed per participant of 2.67 m/s, travelling an average distance of 786.6 ± 3.4 metres on a predetermined outdoor path consisting of various track conditions. The fluctuations in speed, particularly at terrain transitions and street turns, are indicative of the biomechanical demands faced by wheelchair users, such as the need for adjustments during uphill and downhill manoeuvres. These results highlight the importance of real-time movement monitoring in optimising propulsion strategies and reducing user fatigue, ultimately enhancing wheelchair usability and long-term mobility outcomes.

This study builds on previous research while addressing key gaps in real-world wheelchair biomechanics. Prior studies [16,27] have validated IMU sensors for propulsion analysis, with de Vries et al. [27] specifically demonstrating their reliability in real-life wheelchair mobility tracking. However, our findings extend this work by examining how environmental factors influence propulsion speed and biomechanical adaptations in outdoor conditions. While sensor-based propulsion monitoring has been widely explored, our study highlights the need for adaptive propulsion strategies that dynamically respond to terrain variations and user-specific mobility demands. For example, the centre of pressure (COP) shifted considerably throughout the entire test track by 35 mm longitudinally and by 25 mm laterally. This type of movement results in fluctuating and shifting pressure on the seat.

The boxplot shown in Figure 19 illustrates the distribution of R^2^ across all three primary biomechanical domains (speed, COPx, and COPy). This composite visualisation serves as a descriptive behavioural signature for each participant and not as a basis for interferential comparison. It illustrates the full range of individual interaction with the urban environment. The vertical variance (IQR) indicates intra-subject variability and reflects how consistently a participant responded to different route conditions. Remarkably, while participants such as P1 and P3 represent the phenomenological extremes of absolute compliance magnitude, a complementary concordance analysis (Kendall’s W) confirms that these individual rankings remain statistically consistent across independent mechanical and control domains.

Our analysis reveals two distinct dimensions of interindividual variability in urban wheelchair propulsion: magnitude of compliance and cross-domain consistency. For example, while P1 showed the lowest absolute correlation with the route conditions, P5 exhibited the most stable route-dependent behaviour across all investigated parameters. By using Kendall’s W (0.7857) as a complement to the descriptive boxplots, we provide a robust characterisation of these individual behavioural signatures, which holds up despite the limited sample size (*n* = 7) of the pilot study.

### 4.1. Future Perspectives of the Practical Applications of Wheelchair Data

**Figure 20:**
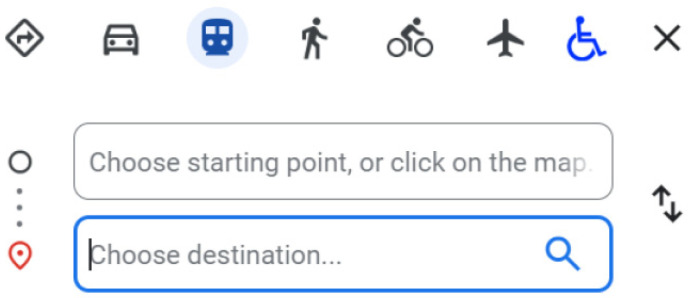
Google Maps real-time route planning search engine, supplemented by a wheelchair symbol (♿, in blue).

The rapid advancement of wearable technology is shaping the future of assistive devices and mobility aids, with a strong emphasis on affordability, artificial intelligence (AI), Internet of Things (IoT) integration, and predictive analytics [45]. These innovations aim to enhance accessibility and independence, ultimately improving the quality of life for individuals with mobility impairments. Continuous wireless data collection through IoT-enabled inertial measurement units (IMUs) enables real-time kinematic data analysis, providing healthcare professionals and researchers with valuable insights into movement patterns and broader population health trends. Integrating these data with navigation platforms such as Google Maps could offer a comprehensive mobility solution for wheelchair users. By leveraging real-time data on terrain conditions, traffic flow, and potential obstacles, such a system could optimise route planning and enhance accessibility. Terrain-specific parameters, including surface smoothness and slope inclination, could be analysed to recommend the most suitable routes for ease of travel. Additionally, predictive analytics could forecast potential mobility challenges, such as traffic congestion or adverse environmental conditions, allowing users to proactively adjust their routes. Crowdsourced data contributions would further refine these recommendations by continuously updating information on accessibility features, hazards, and alternative pathways. The data collected from the sensors (and also from the users’ smartphones) can be used for urban planning, for example, to improve accessibility, remove obstacles, optimise boarding and alighting options in public transport, and mark wheelchair routes in difficult terrain (e.g., inclines). This type of geo-located data could, in the future, be integrated into navigation platforms like Google Maps to provide accessibility ratings for sidewalks and pathways. The corresponding search function in Google Maps (Figure 20) could then be enhanced with a wheelchair icon (♿).

Furthermore, a sudden or gradual decrease in performance may be observed, for example, on the same route but at a significantly reduced speed. This decrease in performance can have various causes. These can be divided into external factors such as weather, traffic (pedestrians and vehicles), or temporary construction sites, and internal factors such as user-related factors. The latter can be due to medical problems (fatigue, joint pain, respiratory illnesses, cardiovascular problems, infections, colds, or the use of new medications) or technical conditions such as a new wheelchair. User-related factors are highly relevant and should be taken seriously. The data could be made available to general practitioners or medical technicians to determine therapeutic measures or to adjust the wheelchair. The latter is particularly important for wheelchair novices who are gradually becoming accustomed to the wheelchair and whose musculoskeletal system is adapting to this new mode of locomotion. The sensor data could determine the optimal time to prescribe a more suitable wheelchair. Overall, this integrated approach seeks to alleviate travel barriers and enhance the mobility experience for individuals with disabilities. The next steps in this research include expanding the study to clinical trials with various, defined wheelchair user groups.

### 4.2. Limitations

While this study provides a high-resolution mapping of wheelchair propulsion in complex urban environments, it also has some limitations. First, the participant sample was limited to able-bodied individuals to validate the system, which may not fully represent the diverse range of biomechanics and propulsion strategies exhibited by wheelchair users with varying disabilities. Future studies should include individuals with different levels of mobility impairment to enhance generalizability. Additionally, while the IMU and GPS data provide valuable insights into outdoor kinematic patterns, the absence of complementary physiological metrics (e.g., muscle activity, heart rate) limits the ability to assess the full impact of propulsion on user fatigue and long-term health outcomes. Addressing these limitations in future research will strengthen the applicability and clinical relevance of smart wheelchair monitoring systems.

Second, the sample size (*n* = 7) is typical for biomechanical pilot studies with intensive real-world data collection. Although this cohort size exceeds the median of comparable studies in this field (two to twelve participants [24,35,36,37,38]), we are aware that it limits the generalisability of our results to the entire wheelchair-using population. Second, the high interindividual variability, particularly the spectrum between route-oriented and individualistic behaviour, suggests that average group responses may mask important individual strategies. To minimise the risk of pseudoreplication and over-inference, we used a descriptive ranking approach and Kendall’s W-concordance analysis instead of purely population-based *p*-values. This enabled us to identify stable patterns of behaviour that persist across multiple parameters, even in a small sample.

Third, since the 13 course segments were completed in a single, continuous trial, serial dependencies in speed and COP position may exist. Our decision to report median effect sizes and rank-based consistency was a deliberate strategy to prioritise robustness over individual-level noise. Future research with larger, more heterogeneous cohorts is needed to confirm whether these individualistic vs. conformist profiles represent distinct clinical phenotypes or a fluid continuum of adaptation. Furthermore, we developed a novel methodological approach to analysing behavioural patterns by introducing a combined assessment tool that includes Absolute Compliance (representing the global magnitude of R^2^) and Domain Consistency (demonstrating trait stability). These two parameters, derived from our pilot study, should be explored in future large-scale studies, particularly those assessing urban accessibility and ranking the difficulty of obstacle overcoming. If a section of a route is affected by a significant obstacle, we expect high scores for both Absolute Compliance and Domain Consistency. These scores, or their rankings, can be integrated into digital city maps for wheelchair users.

Fourth, the inaccuracies of the GPS signal (Figure 4) are a well-known problem caused by high-rise buildings, leading to signal reflections and multipath errors. These inaccuracies are already addressed by automatic correction to the nearest street using smartphone apps (e.g., Google Maps) and vehicle-based navigation systems.

Fifth, to validate the internally developed seat mat sensor array, it was placed on an independent reference device, a force plate, and the COPs of the mat and the force plate were correlated (Figure 2). Bland and Altman explain in detail in their publication [46] why correlation analysis is not suitable for comparing two methods with respect to their degree of agreement. Put simply, if one method is affected by a systematic error and provides measurement data that are a constant higher than those of a reference method, the two methods will still correlate perfectly but will no longer agree. In Bland–Altman diagrams, the limits of agreement must be predefined, based on their acceptance for clinical purposes [46]. In the case of a pressure-sensitive seat mat, the position of the COP is clinically irrelevant, but its trend to move is relevant. Therefore, correlation analysis is the method of choice. The shape of the point cloud in the correlation diagram illustrates typical problems of polymeric piezoresistive sensors. For example, a sensor made of a high-viscosity polymer in the solid state experiences a phase shift of its signal due to excessive electrical viscosity [47], resulting in an elliptical point cloud. Nonlinear point clouds indicate a change in sensitivity across the entire measurement range. The slope of the linear equation of the regression line (ideally Y = 1 × X + 0) reveals disagreements of amplitudes. For example, if the slope is 0.5 instead of the ideal value of 1, one method will only provide half the amplitude of the other; non-zero intercept is irrelevant. When comparing data from the seat mat and the force plate, the higher the R^2^ value, the more accurate one method is compared to the other. When using linear regression, the linearity of the point cloud should be verified. Movement of the centre of gravity (COP) is crucial when sitting in a wheelchair because it results from the variable increase and decrease in pressure on different areas of the buttocks, thus improving blood flow. According to [48], seat pressure should be kept below 20–30 mmHg (2.7–4.0 kPa) to prevent capillary occlusion, particularly at bony prominences such as the ischial tuberosities. A mobile COP maintains the cycle of occlusion and capillary refill with reactive hyperaemia, thereby reducing the risk of pressure sores and ultimately decubitus ulceration. COP mobility is typically achieved by tilting the seat [49,50], or repositioning and using pressure-redistributing support surfaces [51]. A simple example illustrates the effect of pressure redistribution on the COP position. The average distance between the ischial tuberosities is 134.9 ± 9.2 mm in women and 116.5 ± 16.0 mm in men [52]. If body weight is evenly distributed across the ischial tuberosities but concentrated around them, a lateral shift of the centre of pressure by 10 mm leads to a reduction in pressure on one tuberosity and an overload on the other by 7.43% and 8.57% of body weight, respectively, in women and men.

Sixth, since limited sample sizes and interindividual differences can significantly impair the robustness and generalisability of biomechanical studies, advanced analytical methods may be required to draw reliable conclusions from datasets of restricted movements [53].

## 5. Conclusions

This study presents an integrated, sensor-based tracking system for real-time monitoring of wheelchair propulsion and posture. Using IMUs (gyroscopes on the wheels), GPS, and a pressure-sensitive mat on the seat, the system enables detailed biomechanical analysis in the field regarding wheelchair speed and COP shift on the seat. Fluctuations in speed and COP movements correlated well with the different distances travelled and also between participants. The propulsion behaviour, postural adaptation, and usability in wheelchair users with a defined disability remain to be demonstrated in clinical cohorts.

## Figures and Tables

**Table 1:** Legs and landmarks of the route, including distance and average data (velocity, COPx, COPy).

Sections and Landmarks	Description	Terrain Features/Comments	Distance D (m)	ΔD (m)	Velocity (m/s) Avg ± Std	COPx (mm) Avg ± Std	Copy (mm) Avg ± Std
**1**	**Car park, at the back of 6 Luton Lane**	Start of data collection	0	47	0.8 ± 0.23	−1.56 ± 6.02	−17.45 ± 7.94
*1/2*	*Corner car park/Manningtree Road*	right turn					
**2**	**Manningtree Road**	pavement tilted to the left	47	124	1.02 ± 0.25	−4.32 ± 7.59	−18.35 ± 8.03
*2/3*	*Corner Manningtree Road/Guest Street*	right turn					
**3**	**Guest Street**	downhill	171	99	1.26 ± 0.36	−3.79 ± 6.61	−17.64 ± 7.99
*3/4*	*Side street, extension of Luton Lane*						
**4**	**Guest Street**	downhill	270	55	1.43 ± 0.37	−1.52 ± 7.44	−17.35 ± 7.66
*4/5*	*Corner Guest Street/Burwood Road*	right turn					
**5**	**Burwood Road**	narrow pavement due to construction site	325	39	1.04 ± 0.23	−4.21 ± 6.79	−20.83 ± 7.27
*5/6*	*Corner Burwood Road/Cook Street*	right turn					
**6**	**Cook Street**	no pavement, occasional car traffic	364	43	1.05 ± 0.22	2.35 ± 7.64	−23.83 ± 7.26
*6/7*	*Corner Cook Street/Luton Lane*	left turn					
**7**	**Luton Lane**	no pavement, occasional car traffic	407	74	1.4 ± 0.4	5.58 ± 9.17	−20.82 ± 8.61
*7/8*	*obstacle*	defect road surface					
**8**	**Luton Lane**	no pavement, occasional car traffic	481	40	1.28 ± 0.43	4.05 ± 12.29	−21.88 ± 9.51
*8/9*	*Transition from flat to inclined surface*	end of level segment					
**9**	**Luton Lane**	steep uphill, no pavement, occasional car traffic	521	50	0.95 ± 0.25	3.47 ± 8.26	−17.43 ± 15.51
*9/10*	*Corner Luton Lane/Glenferrie Road*	right turn					
**10**	**Glenferrie Road**	downhill, pedestrians	571	98	1.41 ± 0.32	−1.8 ± 7.27	−19 ± 8.19
*10/11*	*Corner Glenferrie Road/Manningtree Road*	right turn					
**11**	**Manningtree Road**	pavement tilted to the left	669	30	1.32 ± 0.23	−4.1 ± 7.29	−20.01 ± 7.93
*11/12*	*obstacle*	small step between 2 pavement plates					
**12**	**Manningtree Road**	pavement	699	48	1.29 ± 0.24	−2.04 ± 8.02	−21.33 ± 7.94
*12/13*	*Corner Manningtree Road/car park*	right turn					
**13**	**Car park, at the back of 6 Luton Lane**	final sprint	747	42	1.81 ± 0.64	1.93 ± 9.03	−19.8 ± 8.1
*finish*		End of data collection	789				

**Table 2:** Medians (med) and interquartile ranges (IQR) of R^2^ data of all participants when correlating their velocity (or COPx, or COPy) with those of the other six participants. The colour coding of the cells reflects the magnitude of the R^2^_med_ data: the larger the R^2^_med_ data, the more strongly the parameters are influenced by the course conditions; the smaller the R^2^_med_ data, the less the parameters are influenced by the course conditions and the more strongly by individual factors; # = number of participant.

Participants	R^2^ of Correlated Velocities		R^2^ of Correlated COPx		R^2^ of Correlated COPy	
#	**R^2^_med_**	R^2^_IQR_	**R^2^_med_**	R^2^_IQR_	**R^2^_med_**	R^2^_IQR_
P1	**0.307**	0.054	**0.334**	0.142	**0.141**	0.104
P2	**0.469**	0.045	**0.226**	0.038	**0.170**	0.205
P3	**0.463**	0.061	**0.273**	0.184	**0.013**	0.166
P4	**0.437**	0.081	**0.287**	0.289	**0.063**	0.030
P5	**0.499**	0.049	**0.425**	0.181	**0.235**	0.161
P6	**0.497**	0.021	**0.406**	0.213	**0.205**	0.151
P7	**0.490**	0.098	**0.109**	0.034	**0.194**	0.220

**Table 3:** Ranks of median R^2^ shown in Table 2 and their rank sum; the colour coding of the cells reflects the ranks.

Participants	Ranks of Mean R^2^ of Correlated Velocities	Ranks of Mean R^2^ of Correlated COPx	Ranks of Mean R^2^ of Correlated COPy	Rank Sum
P1	1	5	3	**9**
P2	4	2	4	**10**
P3	3	3	1	**7**
P4	2	4	2	**8**
P5	7	7	7	**21**
P6	6	6	6	**18**
P7	5	1	5	**11**

## Data Availability

The data presented in this study are available on request from the first author to any qualified researcher who has obtained Ethics Approval for secondary use of existing data through a Consent Waiver.
